# Bulk Anatomical Density Based Dose Calculation for Patient-Specific Quality Assurance of MRI-Only Prostate Radiotherapy

**DOI:** 10.3389/fonc.2019.00997

**Published:** 2019-10-02

**Authors:** Jae Hyuk Choi, Danny Lee, Laura O'Connor, Stephan Chalup, James S. Welsh, Jason Dowling, Peter B. Greer

**Affiliations:** ^1^School of Mathematical and Physical Sciences, University of Newcastle, Newcastle, NSW, Australia; ^2^Department of Radiation Oncology, Calvary Mater Newcastle Hospital, Newcastle, NSW, Australia; ^3^School of Electrical Engineering and Computing, University of Newcastle, Newcastle, NSW, Australia; ^4^South Western Sydney Clinical School, University of New South Wales, Sydney, NSW, Australia; ^5^The Australian E-Health Research Centre, Commonwealth Scientific and Industrial Research Organisation, Brisbane, QLD, Australia

**Keywords:** MRI-only planning, synthetic CT, bulk density, anatomical structure, quality assurance, dosimetric verification

## Abstract

Prostate cancer treatment planning can be performed using magnetic resonance imaging (MRI) only with sCT scans. However, sCT scans are computer generated from MRI data and therefore robust, efficient, and accurate patient-specific quality assurance methods for dosimetric verification are required. Bulk anatomical density (BAD) maps can be generated based on anatomical contours derived from the MRI image. This study investigates and optimizes the BAD map approach for sCT quality assurance with a large patient CT and MRI dataset. 3D T2-weighted MRI and full density CT images of 54 patients were used to create BAD maps with different tissue class combinations. Mean Hounsfield units (HU) of Fat (F: below −30 HU), the entire Tissue [T: excluding bone (B)], and Muscle (M: excluding bone and fat) were derived from the CT scans. CT based BAD maps (BAD_BT,CT_ and BAD_BMF,CT_) and a conventional bone and water bulk-density method (BAD_BW,CT_) were compared to full CT calculations with bone assignments to 366 HU (measured) and 288 HU (obtained from literature). Optimal bulk densities of Tissue for BAD_BT,CT_ and Bone for BAD_BMF,CT_ were derived to provide zero mean isocenter dose agreement to the CT plan. Using the optimal densities, the dose agreement of BAD_BT,CT_ and BAD_BMF,CT_ to CT was redetermined. These maps were then created for the MRI dataset using auto-generated contours and dose calculations compared to CT. The average mean density of Bone, Fat, Muscle, and Tissue were 365.5 ± 62.2, −109.5 ± 12.9, 23.3 ± 9.7, and −46.3 ± 15.2 HU, respectively. Comparing to other bulk-density maps, BAD_BMF,CT_ maps provided the closest dose to CT. Calculated optimal mean densities of Tissue and Bone were −32.7 and 323.7 HU, respectively. The isocenter dose agreement of the optimal density assigned BAD_BT,CT_ and BAD_BMF,CT_ to full density CT were 0.10 ± 0.65% and 0.01 ± 0.45%, respectively. The isocenter dose agreement of MRI generated BAD_BT,MR_ and BAD_BMF,MR_ to full density CT were −0.15 ± 0.90% and −0.16 ± 0.65%, respectively. The BAD method with optimal bulk densities can provide robust, accurate and efficient patient-specific quality assurance for dose calculations in MRI-only radiotherapy.

## Introduction

Magnetic resonance imaging (MRI) only treatment planning is of current interest to reduce systematic registration errors between CT and MRI and improve workflows (1–4). MRI-only treatment planning involves generation of synthetic CT (sCT), since it is not straightforward to convert MRI to electron densities of different tissue classes which are necessary for photon dose calculation in treatment planning systems (TPS).

Different methods have been introduced to create sCT scans for prostate radiotherapy planning. Bulk-density planning was initially investigated as a method for sCT generation (5–10). These studies applied a density of water to the body with an additional separate density for bone. Atlas-based methods involve pair-wise image registration of CT and MRI scans based on anatomical structures to form the atlas, registration of atlas MRI scans to target MRI scan, and mapping the estimated Hounsfield unit (HU) values based on the atlas CT data (11–14). Patch based methods involve feature extraction and patch partitioning from interpatient group-wise affine registration (10, 15). The target feature patches are selected using the approximate nearest neighbor search from the training cohort and sCT patches are generated using the multipoint-wise aggregation scheme. Tissue-classification methods have been developed which assign a single density to each tissue class or assign the continuous HU value based on tissue class probabilities (2). Calibration-type voxel methods use mapping of the MRI signal to HU, however, these require initial identification of bone and surrounding tissue regions with application of separate mapping functions (16). More recently deep learning approaches show promise particularly for generation speed (17, 18). Information on sCT generation methods are available from recent review articles (19, 20).

However, sCT scans are computer generated from large-field-of-view MRI data which can contain artifacts due to image non-uniformities and magnetic field inhomogeneities which can be both scanner and patient dependent (21, 22). They must perform accurately for the variation in patient anatomy that is present in the population and this remains a challenge (23). A recent failure modes and effects analysis (FMEA) of MRI-only planning identified that generation of sCT propagated 46 unique failure modes with 15 failure modes having high risk priority numbers (24). This was significantly more failure modes than the conventional workflow. While CT scanning is a robust and consistent technique, the robustness of sCT is not as high or as well-understood and clinical implementation should proceed with appropriate verifications. Therefore, a quality assurance method that could validate sCT on a patient-specific basis would be desirable. Such a method would ideally fulfill the following criteria: be independent of the sCT method; robust to patient anatomical variations; insensitive to MRI scanner artifacts; efficient to perform; easy to automate; and accurate within clinically acceptable limits.

The bulk-density approach is potentially an ideal candidate to achieve the above criteria for quality assurance of sCT. Most studies that have been performed for bulk-density assignment however have had relatively small patient datasets and assigned arbitrary or literature derived values for the densities (5, 6, 8, 9, 25). Improved agreement to CT dose has been demonstrated with calculation of bone density using effective path-length calculations suggesting that accurate dose calculations are achievable (8).

In this study, we investigate and optimize the bulk-density planning approach to develop a method for patient-specific quality assurance of sCT. Two separate bulk-density methods are investigated with two and three tissue classes, respectively. A large patient cohort of 54 prostate patients is used to measure and determine optimal bulk HU values for the tissue classes that minimize differences with full CT dose calculations. The method is tested using MRI scan assignment of the optimal bulk HU values for the 54 patients following automatic segmentation of bone and body contours. It is referred to here as the bulk anatomical density (BAD) map method.

## Methods

### Patient Data

This study used CT and MR data of 54 prostate cancer patients measured in clinical studies. All data was acquired under ethics board approval with informed consent. Detailed patient data and imaging parameter settings are shown in Table 1. These patient MRI scans were previously acquired for development and validation of sCT generation for MRI-only planning or for a prospective study of MRI-only workflow implementation (26).

**Table 1:** Patients and image acquisition parameters.

**Patients**		**Group 1 (*n* = 39)**	**Group 2 (*n* = 15)**
Age		58 ~ 78 year (69 ± 4.7 year)	58 ~ 83 year (72 ± 6.5 year)
Weight		54 ~ 115.4 kg (87.1 ± 13.2 kg)	62 ~ 122 kg (90.2 ± 17.3 kg)
Imaging year		2012 ~ 2014	2017 ~ 2018
Imaging parameters or sequence	CT	32 patients: GE LightSpeedRT (140 kVp; 2.5 mm slice thickness) 7 patients: Toshiba Aquilion (120 kVp; 2.0 mm slice thickness)	3 patients: GE LightSpeedRT (140 kVp; 2.5 mm slice thickness) 6 patients: Toshiba Aquilion (120 kVp; 2.0 mm slice thickness) 6 patients: Siemens SOMATOM (120 kVp; 2.0 mm slice thickness)
	MRI	Siemens Skyra 3.0 T 3D T2-weighted SPACE (Sampling perfection with application optimized contrasts using different flip angle evolution) sequence Echo time (TE) = 102 ms; Repetition time (TR) = 1,200 ms; Flip angle = 135°; Field of view (FOV) = 430 mm; Slice thickness = 1.6 mm	
Treatment plan		39 IMRT	11 IMRT, 4 VMAT

### Bulk Anatomical Density Maps

**Figure 1:**
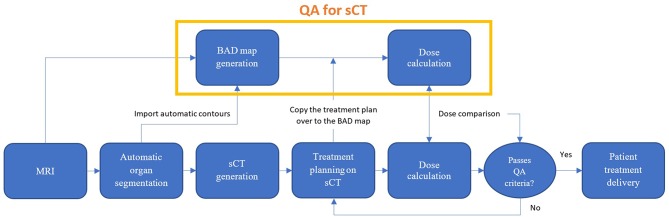
Proposed MRI-only radiation therapy workflow with suggested quality assurance steps for sCT dose verification using the bulk anatomical density (BAD) method.

The role of the BAD map in quality assurance of the MRI-only workflow is shown in Figure 1.

**Figure 2:**
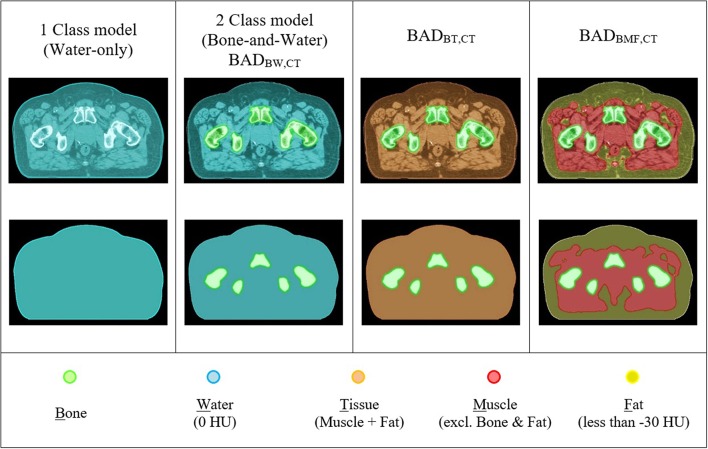
CT based BAD maps. Colors represent the bulk-density assignments on different structures.

The BAD method is an extension of the conventional bulk-density map. Up to three tissue classes have been investigated (Bone, Muscle, and Fat). BAD maps can be made with different tissue class combinations by assigning the bulk HU values to either CT or MRI patient scans; (1) BAD_BW_ (Bone and Water), (2) BAD_BT_ (Bone and the entire Tissue), and (3) BAD_BMF_ (Bone, Muscle, and Fat). The BAD methods are compared to the conventionally used methods and are shown in Figure 2. Derivation of the method has been performed using CT scan data.

### Mean Density of Tissue Classes

Tissue segmentation was performed on CT scans to determine mean HU values. CT images were segmented into different tissue classes based on the HU values: Bone (B: >100 HU); Fat (F: HU below −30 HU), and the entire tissue (T: including Fat and Muscle but excluding Bone) areas. Muscle area (M: excluding Bone and Fat) was found using a Boolean operation of [*B*^*c*^ ∩ *F*^*c*^] within body. These were performed using the automatic contouring tools of Varian Eclipse (Varian Medical Systems, Palo Alto, CA, USA). According to Kim et al., HU of adipose tissue, including both subcutaneous and visceral, is within a range of −140 to −30 HU (27). Note that, the Muscle volume includes other organ structures such as bladder and rectum (with gas/air).

The population average mean HU values (±1 standard deviation) of each structure were; Bone = 365.5 ± 62.2 HU, Fat = −109.5 ± 12.9 HU, Muscle = 23.3 ± 9.7 HU, the entire Tissue (T) = −46.3 ± 15.2 HU. Using the department's HU to electron-density conversion curve within the Eclipse TPS these HUs are corresponding to the relative electron densities of 1.17 (Bone), 0.91 (Fat), 1.03 (Muscle), and 0.97 (the entire Tissue) and physical densities of 1.23 g/cm^3^ (Bone), 0.92 g/cm^3^ (Fat), 1.04 g/cm^3^ (Muscle), and 0.98 g/cm^3^ (the entire Tissue).

### Derivation of Optimal Densities

To determine the optimal bulk-density values a linear fitting method was employed with the assumption that the dose change is approximately proportional to tissue density change at least over small ranges. The BAD_BW,CT_, BAD_BT,CT_, and BAD_BMF,CT_ were created using the mean values for the tissue classes as determined above. Additionally they were also generated with a separate bone value of 288 HU that was derived using effective path lengths by Lambert et al. (8) and the equation presented by Thomas (28). Assigned densities were rounded up since fractional values are not accepted on planning system (Varian Eclipse).

Here, 6 separate BAD maps were created as follows: BAD_BW,CT_ [Bone = 288 HU; Tissue = 0 HU (Water)], BAD_BT,CT_ (Bone = 288 HU; Tissue = −46 HU), BAD_BMF,CT_ (Bone = 288 HU; Muscle = 23 HU; Fat = −109 HU), and BAD_BW,CT_ [Bone = 366 HU; Tissue = 0 HU (Water)], BAD_BT,CT_ (Bone = 366 HU; Tissue = −46 HU), BAD_BMF,CT_ (Bone = 366 HU; Muscle = 23 HU; Fat = −109 HU). IMRT treatment plans previously developed on the corresponding patient CT or sCT scans were then copied to the BAD maps and dose was recalculated on the BAD maps using the same monitor units and fluences. The same plan dose was also calculated on the gold-standard CT scan.

**Figure 3:**
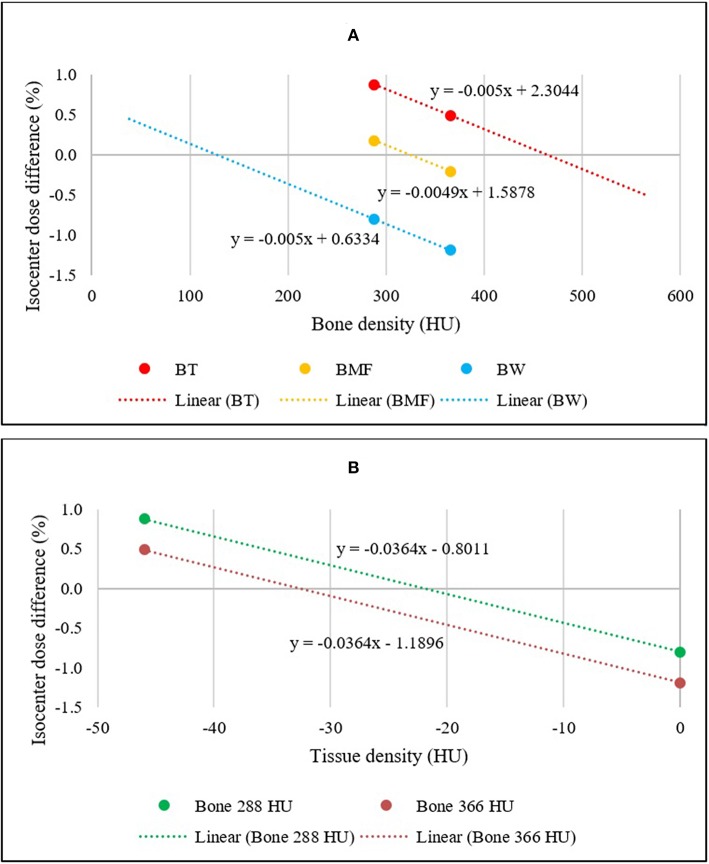
**(A)** Linear relationships between the mean isocenter dose differences of BAD_BW,CT_, BAD_BT,CT_, and BAD_BMF,CT_ maps to full density CT for two bone density values. The x-intercepts represent the optimal bulk bone density of each method. **(B)** Linear relationships between the mean isocenter dose differences of BAD_BW,CT_ and BAD_BT,CT_ maps to full density CT. The x-intercepts represent the optimal bulk Tissue density for BAD_BT,CT_.

The mean differences in dose to isocenter for the BAD maps and the CT scan of all 54 patients were determined and plotted. Figure 3A illustrates a linear plot for each BAD map method with bone density as the x-axis. This plot can be used to determine an approximately optimal bone density for the methods using the intercept for zero mean dose difference to CT. The calculated optimal bone densities for BAD_BW,CT_, BAD_BT,CT_, and BAD_BMF,CT_ were approximately 127.2, 463.8, and 323.7 HU, respectively. The averaged measured mean density of bone was 365.5 ± 62.2 HU, and therefore the derived optimal bulk-density of bone value of 324 HU (rounded up) was subsequently only used for the BAD_BMF_ maps, and the measured value of 366 HU (rounded up) was retained for the BAD_BW_ and the BAD_BT_ maps.

Figure 3B shows similar plots for the BAD_BW,CT_ and BAD_BT,CT_ maps but with the x-axis changed to the Tissue values. These maps are analogous in that the anatomical regions used are identical. This allows for determination of the optimal density for Tissue for BAD_BT,CT_ maps from the intercept for zero mean dose difference. The calculated optimal densities of the Tissue were approximately −22.0 and −32.7 HU with bone densities of 288 and 366 HU, respectively. For subsequent BAD_BT_ maps the measured bone value of 366 HU was used and therefore the tissue density of −33 HU (rounded up) was adopted i.e., BAD_BT_ (Bone = 366 HU; Tissue = −33 HU). HU of 324 is corresponding to relative electron densities of 1.16 and physical densities of 1.20 g/cm^3^ while HU of −33 is corresponding to relative electron densities of 0.97 and physical densities of 0.99 g/cm^3^.

### Dosimetric Accuracy for Optimal BAD_CT_ Maps

Using the optimal densities and CT derived anatomical contours, two BAD_CT_ maps [BAD_BT,CT_ (Bone = 366 HU; Tissue = −33 HU) and BAD_BMF,CT_ (Bone = 324 HU; Muscle = 23 HU; Fat = −109 HU)] were created and tested for all 54 patients. As described above, doses were recalculated on these BAD maps and compared to CT calculation. Isocenter doses of each plan were compared to the corresponding CT dose.

### Dosimetric Accuracy for BAD_MR_ Maps

**Figure 4:**
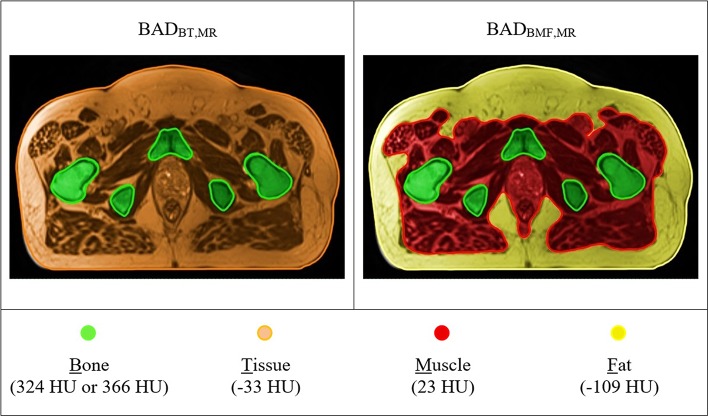
T2-weighted MR based BAD maps with optimal density values. Colors represent the bulk-density assignments on different structures. BAD_BT,MR_ (Bone, B = 366 HU; Tissue, T = −33 HU) and BAD_BMF,MR_ (Bone, B = 324 HU; Muscle, M = 23 HU; Fat, F = −109 HU).

The method was then applied to the large-field-of-view T2-weighted MRI scans for the patients. Two BAD_MR_ maps [BAD_BT,MR_ (Bone = 366 HU; Tissue = −33 HU) and BAD_BMF,MR_ (Bone = 324 HU; Muscle = 23 HU; Fat = −109 HU)] were created analogous to CT above (Figure 4). To derive the anatomical contours for density assignment, the automatic MRI body and bone contouring method that was developed in a previous sCT study was utilized (13). For the BAD_BMF,MR_ the fat contour created on the anatomically (rigid) registered CT was used for density assignment. This will require replacement with an MRI based method in the future, for example using DIXON scans, however these were not available at this time. It is assumed that the segmentation of fat in MRI will correspond to the fat utilized here. The treatment plan on CT was copied directly over to the BAD_MR_ maps and isocenter doses of each map were compared with the corresponding CT plan. The 3D Gamma comparison metric was used for dose comparisons in all voxels (29). This used varying gamma criteria 3%, 3 mm, 2%, 2 mm, 1%, 1 mm, a low dose threshold of 20% for inclusion, and the CT dose was used as the reference. A 15 mm erosion operator was used to remove the region close to the skin border from the calculation as this gives large gamma discrepancies due to contour differences.

## Results

### Dosimetric Accuracy for BAD_CT_ Maps Using Measured Densities

**Figure 5:**
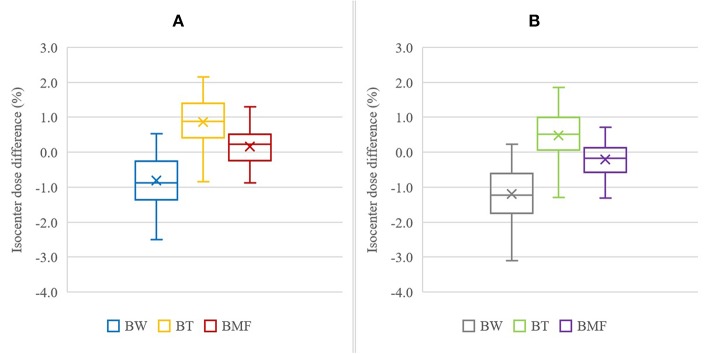
Isocenter dose difference to CT for BAD_CT_ methods using measured (non-optimal) densities with bone, B, as 288 HU **(A)** and 366 HU **(B)**. The other tissue densities used were: Water, W = 0 HU; Tissue, T = 46 HU; Muscle, M = 23 HU; and Fat, F = −109 HU. The cross mark “×” represent the mean of the results, the horizontal bar inside the box is the median, the extent of the boxes represents the interquartile range (IQR) between the first quartile (Q1) and the third quartile (Q3), and the ends of whiskers represent the minimum and maximum range.

The isocenter point dose results are shown in Table 2 and Figure 5 for the six BAD_CT_ maps for measured (non-optimal) mean HU values and the same maps but with bone density of 288. The BAD map with three tissue classifications (BMF) provided the closest matching to the full density CT plans with smaller variations compared to other two bulk-density maps (BW and BT). However, significant systematic differences to CT are still evident particularly for the first two methods. The literature bone density performs slightly better for the BW map while the measured mean bone density performs better for the BT map while there is no clear winner for the BMF map.

**Table 2:** Mean (±1 standard deviation) isocenter dose difference for BAD maps from full density CT plan using measured (non-optimal) densities (Water, W = 0 HU; Tissue, T = 46 HU; Muscle, M = 23 HU; and Fat, F = −109 HU) and two bone densities, measured (B = 366 HU) and literature (B = 288 HU).

**BAD_**CT**_ plan**	**Isocenter dose difference to CT (%)**	
	**B = 288 HU**	**B = 366 HU**
BW	−0.80 ± 0.69	−1.19 ± 0.69
BT	0.87 ± 0.63	0.49 ± 0.63
BMF	0.18 ± 0.45	−0.21 ± 0.45

### Dosimetric Accuracy for Optimal BAD_CT_ Maps

**Figure 6:**
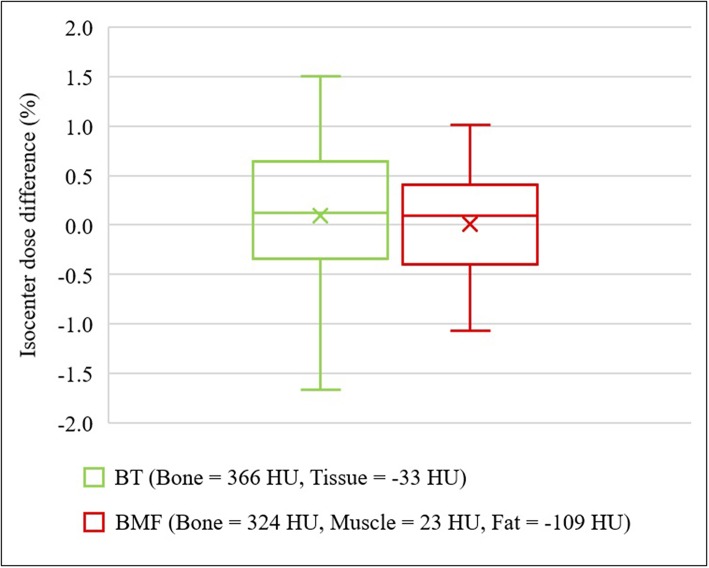
Isocenter dose differences to CT for optimal density BAD_CT_ maps. BAD_BT,CT_ (Bone, B = 366 HU; Tissue, T = −33 HU) and BAD_BMF,CT_ (Bone, B = 324 HU; Muscle, M = 23 HU; Fat, F = −109 HU).

Figure 6 shows the results for two optimal density BAD_CT_ maps [BAD_BT,CT_ (Bone, B = 366 HU; Tissue, T = −33 HU) and BAD_BMF,CT_ (Bone, B = 324 HU; Muscle, M = 23 HU; Fat, F = −109 HU)]. Significant improvements were observed for both optimal BAD maps and the isocenter dose differences to CT were observed as 0.10 ± 0.65 and 0.01 ± 0.45%, respectively. The interquartile ranges (IQR) were from −0.34% to 0.65% and −0.39% to 0.41%, respectively.

### Dosimetric Accuracy for BAD_MR_ Maps

**Figure 7:**
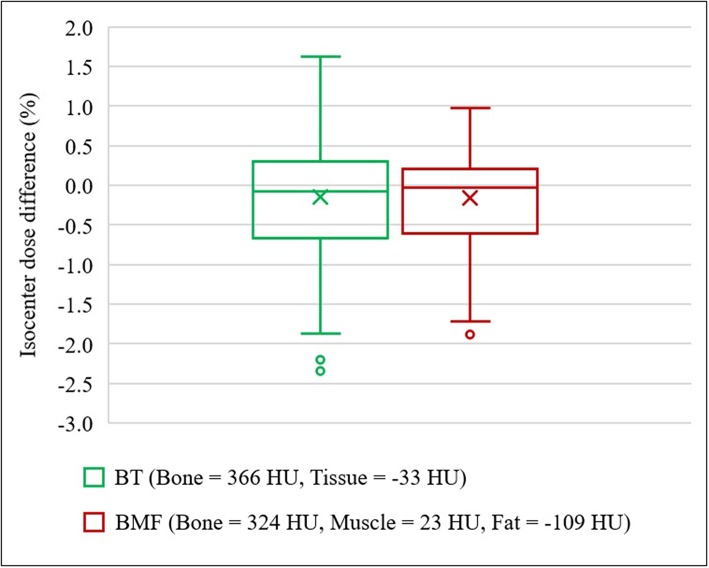
Isocenter dose differences to CT for optimal density BAD_MR_ maps. BAD_BT,MR_ (Bone, B = 366 HU; Tissue, T = −33 HU) and BAD_BMF,MR_ (Bone, B = 324 HU; Muscle, M = 23 HU; Fat, F = −109 HU). In the presence of outliers, the ends of whiskers indicate the lower (Q1 – 1.5 × IQR) and upper (Q3 + 1.5 × IQR) limits.

With the optimal density assignment, the isodose differences to full density CT plan were observed to be −0.15 ± 0.90% on BAD_BT,MR_ and −0.16 ± 0.65% on BAD_BMF,MR_ (Figure 7). The IQR of both BAD_MR_ maps were within ±0.7%; BAD_BT,MR_ was from −0.65 to 0.31%, while BAD_BMF,MR_ was from −0.60 to 0.22%. The mean differences and standard deviations are slightly larger than the CT derived BAD maps which would be expected due to the different anatomical contours used for MRI. Results of gamma analysis pass-rate are shown in Table 3.

**Table 3:** Gamma analysis pass-rate results for comparison between BAD_MR_ maps and CT dose calculations.

	**BAD**_****BT, MR****_			**BAD**_****BMF, MR****_		
	**3%/3 mm**	**2%/2 mm**	**1%/1 mm**	**3%/3 mm**	**2%/2 mm**	**1%/1 mm**
Mean (%)	99.9 ± 0.2	99.3 ± 1.1	90.7 ± 10.2	99.9 ± 0.2	99.6 ± 0.6	93.8 ± 8.6
Min (%)	99.1	94.6	58.8	99.3	97.2	58.5

*BAD_BT,MR_ (Bone, B = 366 HU; Tissue, T = −33 HU) and BAD_BMF,MR_ (Bone, B = 324 HU; Muscle, M = 23 HU; Fat, F = −109 HU)*.

## Discussion

In this study, the BAD method was developed to map bulk densities to anatomical structures using data measured from 54 prostate cancer patients. A linear interpolation method was used to determine the optimal HU values to give approximately zero mean isocenter dose agreement to CT. Using the optimal HU values, an improvement in isocenter dose agreement was observed when compared with using measured mean HU values. Particularly, the three tissue class BAD_BMF_ method provided the closest dose agreement to conventional CT but the two class BAD_BT_ still gave acceptable agreement. Both optimal bulk-density assigned BAD_MR_ maps provided mean dosimetric differences within 0.2% to conventional CT with standard deviations within 0.9%. Therefore, both methods can be considered for dose verification and patient-specific quality assurance of sCT scans. To use the BAD_BT_ with MRI-only workflows only requires that the body and bone contours on a large-field-of-view MRI scan are segmented and this can be automated with atlas or similar methods. The generation of the BAD map could potentially be fully automated. It meets the criteria outlined above for a method for quality assurance of sCT (independence; robustness; insensitive to scan artifacts; efficient and easy to automate; and accurate within clinically acceptable limits). In principle, the results here also suggest that this method may be adequate in accuracy for sCT generation for dose calculation in MRI-only workflows however these scans may not be suitable for image-guidance.

The linear interpolation method used makes the assumption that the isocenter dose is a linear function of the particular tissue density that is modified. However, this is clearly an oversimplification of dose deposition processes and this can be seen in the results. The optimal densities do not result in exactly zero dose difference to CT dose for the optimal BAD_CT_ maps although the difference is within 0.1%. To obtain a mean of zero an iterative process could be conducted although this would be extremely time consuming for negligible benefit. Furthermore, when applied to MRI scans where the bone contours are derived using an entirely different method, the mean isocenter dose to CT is modified further with a decrease in the BAD map doses to isocenter for the same patients when compared to CT. This could be due to the MRI bone contour being larger than the CT bone contour which is often observed in clinical practice although other factors could also contribute including the body contour.

The methodology was derived using HU which corresponds to the sCT literature. The Eclipse TPS converts these to relative electron density (RED) using our in-house conversion and therefore the RED values as well as physical density are stated in the manuscript.

These results can be compared with other bulk-density methods reported on previous studies. The early reported works in MRI-only planning used bulk-density assignment to CT or MRI but with limited datasets, and in some cases homogeneous CT calculations (5–7). Kim et al. used bulk-density assignment to water and bone for 15 prostate patients with 300 HU assigned to bone that was measured as the mean value within femoral head contours on CT. The PTV (D_95%_) differences were 1.9% (9). Lambert et al. studied 39 prostate patients and for their CT dataset with a density of water and a density of 1.19 g/cm^3^ for bone found a mean dose isocenter difference to CT of 0.2% which is lower than that found here using the same density value (8). The reason for this is not entirely clear, it could be related to changes in TPS calculation algorithms. Their paper used two separate planning systems and dose calculation algorithms have since improved.

There is general lack of consensus on the density to assign to bone and as we have shown here consideration of three tissue classes yields better results. The optimal density is likely to be influenced by the method used to determine the anatomical contour, the method used to determine the optimal density (mean or path length), the volume and location of the anatomical structure, i.e., all bone or just femur, and the TPS algorithm. In many cases these details are not currently given in the relevant literature. An assessment of the sensitivity of bulk-density planning to these factors would be of interest. When the method derived here was applied to MRI data using a different bone contouring algorithm the results were similar which suggest that it is relatively robust to the segmentation method and variations in the bone contour. The method is simple to perform using standard anatomical contouring techniques and TPS system reporting of mean densities to these contours. Therefore, the method can be used to determine an optimal density for any particular center's practice if necessary.

This study used a large patient dataset of 54 CT scans to determine the optimal HU values and validation with 54 patient large-field-of-view MRI scans. Both optimal bulk-density values for bone and the entire tissue are within the variations of the average mean bulk-density calculated from the cohort. Thus, these would be valid for future applications particularly for those male patients weighing between 54 and 122 kg. A consideration would be to apply these values to an external patient CT/MRI dataset for further validation.

One limitation of the study is that the fat contour from the registered CT scan was used for generation of the BAD_BMF,MR_ map. For fat class segmentation on MRI, fast DIXON scans could be incorporated into the MRI acquisition protocol for future study to generate three-class BAD_MR_ maps to improve the dose calculation accuracy, in particular to reduce the standard deviation of the results when compared to CT dose.

Application of automated bone segmentation on MRI may cause dosimetric inaccuracy for the BAD_MR_ maps. More accurate segmentation can potentially be achieved via manual contouring however this is time consuming and the level of accuracy can vary depending on the level of expertise and experience of individuals (10, 30).

Previous studies have demonstrated the accuracy of the atlas-based automatic segmentation method that was used for this study. The automated bone contours had mean Dice similarity coefficient scores of 0.91 ± 0.03 and the mean absolute surface distance of 1.45 ± 0.47 mm when compared to expert drawn manual contours (14). Automatic MRI bone segmentation has become an important component of many sCT generation methods due to its efficiency and accuracy. Korhonen et al. and Koivula et al. also used an atlas-based method for their dual model method for sCT generation and the average PTV mean dose differences of their sCTs to CT were 0.3 ± 0.2% and −0.6 ± 0.4%, respectively (16, 31). Currently, commercially available sCT generation products, for example the FDA-approved Philips Magnetic Resonance for Calculating Attenuation (MRCAT) software package, use a model-based segmentation method for delineating bony structures from the patient's body outline from mDIXON water, fat, and in-phase images (23, 32).

Dose calculation on BAD map could be improved if the density of gas within the rectum was considered. However, most centers routinely control the magnitude of rectal gas through patient preparation prior to scanning or voiding and rescanning. In many cases. the gas may be atypical of treatment and therefore the dose calculation may not reflect treatment dose. Some centers override the density of gas in the rectum for dose calculations. This is a general problem for radiotherapy planning and can be managed in the same way as conventional CT based planning with the advantage that for MRI-only treatment planning rescanning does not require additional patient dose.

In summary, the BAD map is a technique that utilizes anatomical structures for generating BAD maps for patient-specific dose calculations to compare to sCT. With the optimal density assignments, it provides clinically acceptable dose agreement to the conventional full density CT based plans. The three-class BAD model (Bone, Muscle, and Fat) performs best however the two-class BAD model (Bone, Tissue) is also acceptable. The BAD method can provide accurate dose calculations for verifying sCT for clinical use in MRI-only workflows. It has currently been implemented as a quality assurance method in a multi-center trial of prostate stereotactic radiation therapy (NINJA) that includes an MRI-only sub-study.

## Data Availability Statement

The datasets generated for this study are available on request to the corresponding author.

## Ethics Statement

The studies involving human participants were reviewed and approved by Hunter New England Human Research Ethics Committee. The patients/participants provided their written informed consent to participate in this study.

## Author Contributions

JC performed overall data analysis, drafting and editing the manuscript, and manuscript submission. DL assisted with data analysis, contributed to review draft and editing. LO'C assisted with treatment planning and dose calculation. JD calculated MRI automatic bone and body contouring. SC and JW assisted with project design, data analysis, manuscript review and editing. PG assisted with project development, ethical submission, methodology, data analysis, literature review, manuscript review and editing.

### Conflict of Interest

The authors declare that the research was conducted in the absence of any commercial or financial relationships that could be construed as a potential conflict of interest.
